# Efficiency of different treatment modalities on radiation induced trismus for maxillofacial cases: a parallel randomized clinical trial

**DOI:** 10.1186/s12903-025-05600-7

**Published:** 2025-03-04

**Authors:** Marwa Ahmed Aboelez, Abdallah Mohammed Ibrahim, Mohammed A ElSawy, Nermeen El sayed El-Khamisy

**Affiliations:** 1 Faculty of Dentistry, Department of Prosthodontics, Mansoura University, 1 El Haramine St. from Talkha, Mansoura, Egypt; 2 Department of Prosthodontics, Menoufia University, Shibin Al Kawm, Egypt

**Keywords:** Low-level laser, Radiation therapy, Threaded tapered screw appliance, Trismus

## Abstract

**Background:**

For more than 80% of patients with head and neck cancer, radiation therapy (RT) is a crucial component of their treatment plane which causes impairment for the masticatory apparatus functions leading to trismus. The study objective was to compare the efficacy of different treatment modalities for patients with RT-induced trismus on maximum mouth opening (MMO), visual analogue scale (VAS) (primary outcomes) patient satisfaction (secondary outcome).

**Methods:**

Thirty-six patients with trismus after radiation therapy were classified equally and randomly into three groups (*n* = 12 per group): Group A was given threaded tapered screw appliance therapy (TTSA), Group B was given low-level laser therapy (LLLT), and Group C was given both threaded tapered screw appliance and low-level laser therapy (LLLT + TTSA). Maximum mouth opening (MMO), visual analogue scale (VAS) and Gothenburg Trismus Questionnaire (GTQ) scores and time required to achieve normal state were evaluated at baseline, 1, 2, 4 weeks, 3 and 6 months after the intervention. Data were collected and analysed using SPSS software.

**Results:**

Regarding VAS and MMO, there was a statistically significant difference at different times of evaluation within all groups where (*P* <.0001). Regarding GTQ, group C recorded the least values for GTQ symptoms followed by group B followed by group A. Between groups A, B, and C at six months, there was a statistically significant difference for VAS. At three and six months, there was a statistically significant difference between all groups for MMO. At three and six months, there was a statistically significant difference between groups for all GTQ domains.

**Conclusion:**

All available therapy modalities have the potential to effectively improve radiation induced trismus; however, the combination of TTSA and LLLT group appears to yield the most rapid and optimal enhancement.

**Clinical Trial Registry Number:**

(NCT06413628) (05/12/2024) Retrospectively registered.

## Background

A patient’s quality of life is frequently negatively impacted by oral cancer, which is a serious condition. Treatment for oral cancer, which includes radiation, chemotherapy, and surgery, can result in a number of oral complications, including trismus, xerostomia, dyspepsia, odontogenic infections, and osteoradionecrosis [1]. For more than 80% of patients with head and neck cancer, radiation therapy (RT) is a crucial component of their treatment plan, regardless of whether they receive chemotherapy, surgery, or both [2]. Impaired masticatory apparatus functions, which lead to trismus, are among the most difficult late problems in the head and neck region to treat [3].Fibrotic alterations and associated contracture in the masseter and pterygoid muscles, along with injury to their neural supply, vascular system, and temporomandibular joint (TMJ), can result in RT-induced trismus (RIT) [4].

Trismus is characterized by significantly restricted mouth opening that makes it difficult to talk, swallow, bite, or chew. It is observed in objective measures, when the maximum incisal opening (MIO) is less than 35 mm. This leads to a poor quality of life as well as a decreased chance of surviving at all [5, 6]. The severity of trismus can vary from a mild restriction of mouth opening to a complete incapacity to move the jaws, depending on the extent of damage that therapy has inflicted to the masticatory system [7]. Restrictions in mouth and TMJ motions associated with RIT may also hinder speech and social communication abilities in those patients. Moreover, brushing will be extremely difficult, raising the risk of poor oral hygiene and the diseases periodontal disease, dental caries, and tooth infections [8]. If trismus is not treated, it may get worse and cause the patient to lose too many nutrients, rendering them unable to function [8].

Many of the effects of RT-induced fibrosis can be avoided or reduced with early intervention [9]. Treatment objectives are to remove oedema, soften and stretch the fibrous tissue, improve muscular strength, restore circulatory efficiency, thus increasing mouth opening, and retain muscular dexterity [10]. Surgery is frequently used to correct trismus in order to eliminate postoperative complications. But, due to a history of radiation therapy in the region, the patient’s reluctance to have surgery, and financial limitations, second surgeries to relieve trismus in cancer patients are typically avoided. Painful and dangerous side effects can result from forced mandibular opening used to treat trismus. Additionally, because the treatment is very unpleasant, general anaesthesia is required [11].

To prevent severe trismus, most patients receive conservative treatment with conservative tools and instructions [12]. Literature review demonstrated the efficacy of different trismus appliances to improve the mouth opening [13]. Trismus devices can apply force that is light or heavy, elastic or inelastic, and continuous or intermittent manner. Trismus appliances can be classified as either internally or externally actuated depending on how they are designed [14]. While externally activated appliances employed the patient’s depressor muscles to improve mouth opening, internally activated appliances use the patient’s depressor muscles to extend the elevator muscles. It has been demonstrated that the force produced by elevator muscles is ten times more than that of depressor muscles [15].The strength and motivation of the patient determine how much force is used [16]. They consist of tongue blades, a continuous dynamic jaw extension device, a threaded tapered screw, a dynamic bite opener, and a screw-type mouth gag [9].Tongue depressors are the least expensive alternative; they have been used for many years to mobilize the jaw, but they have not been shown to be beneficial and may put an excessive amount of strain on the teeth [17].

Compared to other appliances, threaded tapered screw appliance (TTSA), is less expensive and is simple for the patient to use it. The success of this type of device is mostly dependent on patient motivation. To gradually wedge the teeth apart, the patient inserts the screw between his posterior teeth [18].The patient regulates the amount and timing of pressure needed to progressively widen the gap between the jaws while the threads guide the teeth along the taper [19].

Another effective conservative approach that has gained popularity for treating trismus is low-level laser therapy (LLLT). As a therapy modality, LLLT is a well-accepted by patients and is cost effective, non-invasive, painless, safe, and well-tolerated. Also, it is a quick process that requires little time spent in the dentist’s chair [20]. Low level laser treatment (LLLT) helps the tissue heal by reducing pain and swelling and reducing inflammatory conditions without having negative side effects [21]. The force of LLLT in treating pain originating from soft-tissue trauma can be attributed to the indirect reduction of edema, bleeding, neutrophil activity, provocative cytokines and enzymatic action. LLLT reduces swelling and subsequent pain resulting in, enhanced tissue repair since lymphatic vessels regeneration is accelerated and the vascular permeability is minimized [21].

Although a number of studies [19, 22, 23], have evaluated the effectiveness of threaded tapered screw appliance therapy (TTSA) alone or low-level laser therapy alone for patients with RIT, the literature lacks clinical studies that targeted combination between both modalities. Therefore, this study aimed to compare the efficacy of different treatment modalities for patients with RIT on patient satisfaction and experience. This method was supposed to be more effective in managing RIT conservatively by avoiding more invasive surgical techniques. It was anticipated that the combination of TTSA and LLLT would result in a more significant reduction in clinical signs and symptoms compared to the use of either treatment alone. For patients with RIT, the null hypothesis for this clinical trial was that there would be no differences between the aforementioned therapy modalities in terms of patient satisfaction, maximum mouth opening, and visual analogue scale of pain.

## Methods

### Study design

**Fig. 1:**
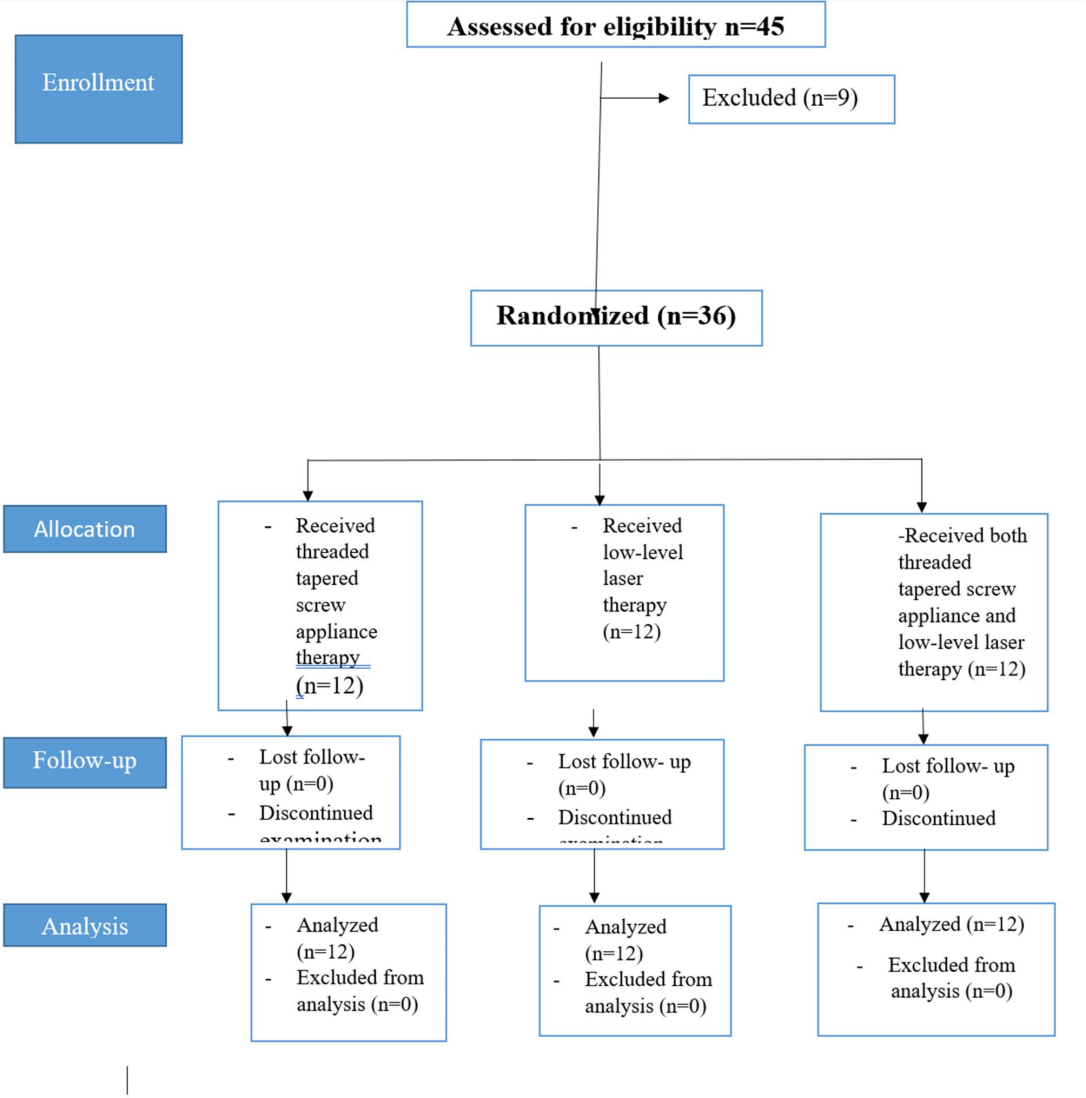
Flow chart

Forty-five patients with trismus and pain after HNC who had radiation therapy were included in the study between August 2022 and December 2023 (twenty females and twenty-five men), and thirty-six of them (sixteen Females, twenty males) completed a parallel randomised clinical trial. Figure 1 displays the study procedures’ flowchart. They arrived at the Maxillofacial unit of the Prosthodontics clinic. The study’s procedures were carried out at Maxillofacial unit in the prosthodontic department. All experiments were performed in accordance with relevant guidelines and regulations. Faculty of dentistry ethics board committee approved this clinical investigation, and it was given the ethical approval number A34080622. The study’s protocol was registered in clinicaltrials.gov (No. NCT06413628). All experiments were performed in accordance with relevant guidelines and regulations.Patients gave their informed consent to participate after learning about the treatment procedures. The study objective was to compare the efficacy of different treatment modalities for patients with RT-induced trismus on patient satisfaction and experience. Based on the results of an earlier investigation [24] where the authors discovered a significant difference in (α = 0.05), this patient sample was estimated using G*Power software (version 3.1.5, Kiel, Germany) to yield 80% power.

## Data collection

The inclusion criteria involved patients with head and neck cancer who received radiation therapy and whose clinical evaluations revealed that they experienced pain and trismus (a maximal incisal opening of less than 35 mm) (b) impaired mastication (c) stiffness/fatigue in jaw (d) pain or soreness in jaw muscles. The exclusion criteria were (a) neurological condition impacting the facial nerve as Bell’s palsy, and (b) sensitivity to phototherapy (c) patients still receiving radiation. Participants were classified equally and randomly into three groups (*n* = 12 per group) using random numbers generated in an Excel spreadsheet. **Group A** was given threaded tapered screw appliance therapy (TTSA), **Group B** was given low-level laser therapy (LLLT), and **Group C** was given both threaded tapered screw appliance and low-level laser therapy (LLLT + TTSA). Randomization and assignment of patients to treatment groups was carried out by dental experts who were not informed about the treatment groups.

**Fig. 2:**
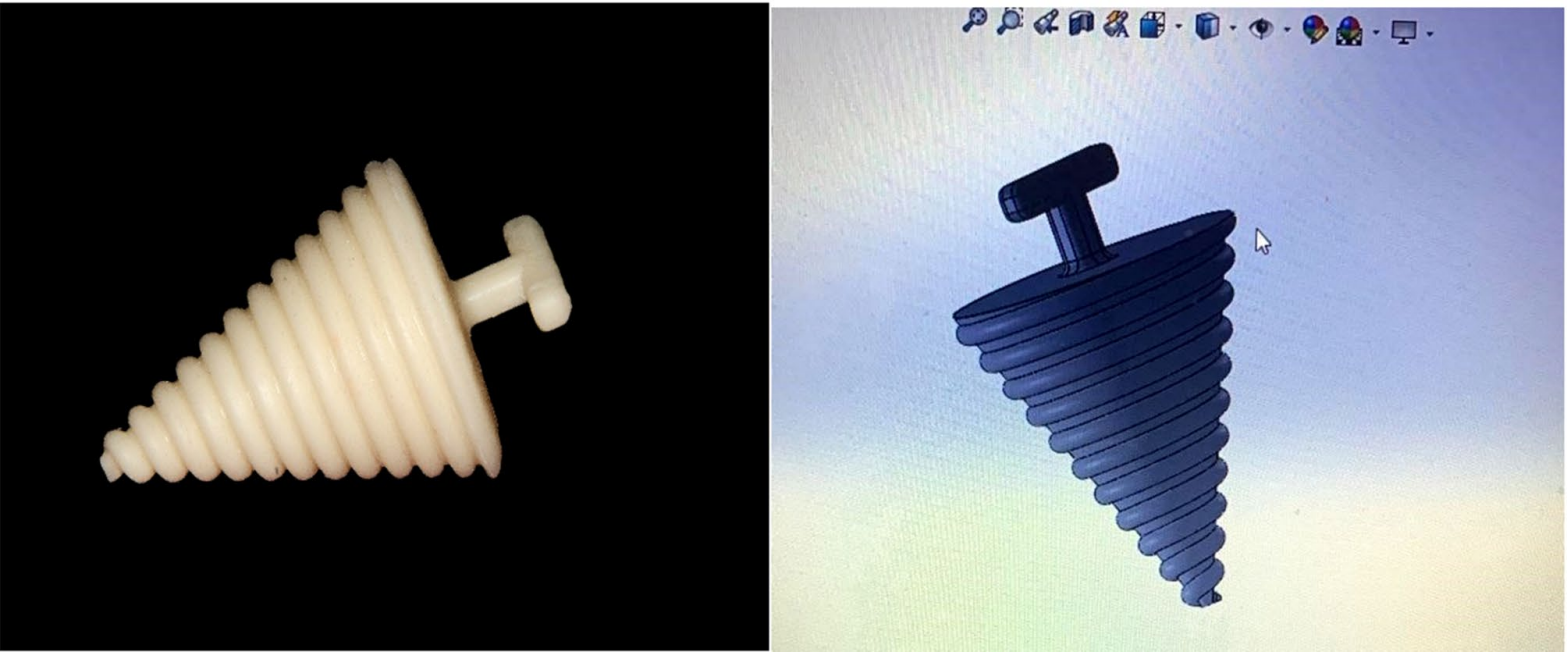
Threaded tapered screw appliance

**Fig. 3:**
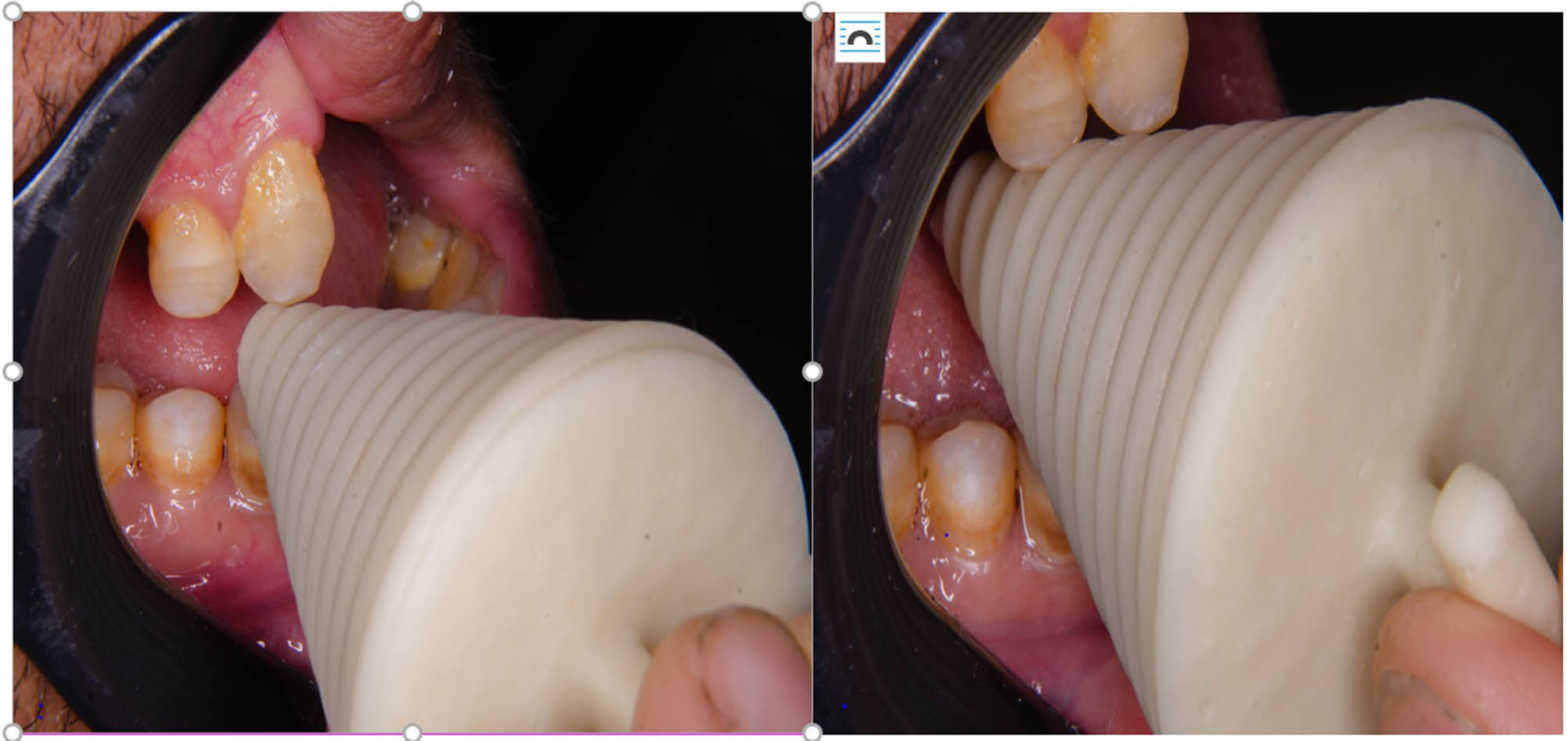
**a**. Intraoral application of the appliance (Before) **b**. Intraoral application of the appliance ( After)

**Group A**, was given threaded tapered screw appliance therapy (TTSA). 3rd CAD design software was utilized to digitally design the appliance using Solid Works tools. (www.solidworks.com) to have the STL file format. The virtual design of was approved. Materials for a 3D-printed denture base (Dentca, denture base) were loaded into the printer (Rasdent 3D printer). The appliance was printed Fig. 2. The patient was instructed to insert (TTSA)’s smaller end between their premolars and use the handle to spin it clockwise. The appliance began to push more lingually, progressively increasing mouth opening. The patient was instructed to perform this exercise two or three times a day Fig. 3.

**Fig. 4:**
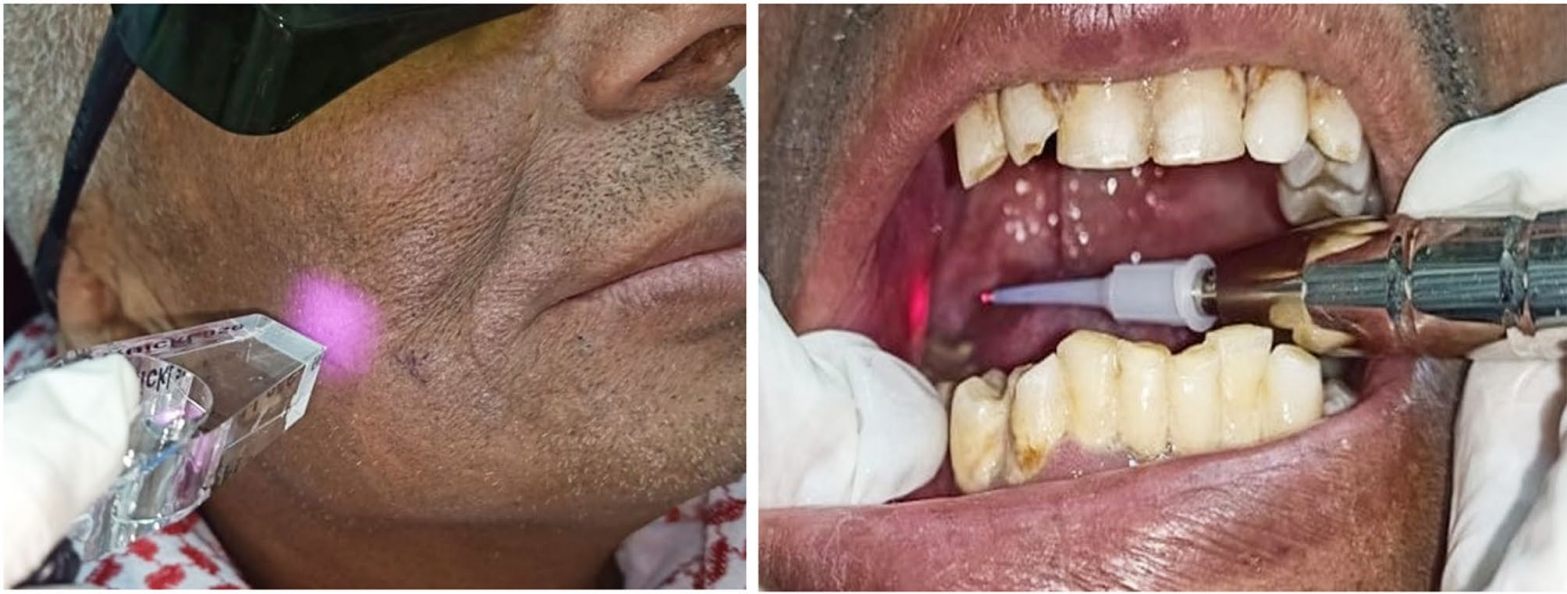
**a**. Introral application of laser beam **b**. Extraoral application of laser beam

**Group B**, was given low-level laser therapy (LLLT), used laser was a diode laser (quicklase, Britain). Laser device comprised a hand-piece equipped with 2 options: a plastic tip for delivering laser energy inside the mouth when targeting the pterygoid muscles, or a custom optic prism attached to the tip for applying laser externally to extraoral muscles Fig. 4. This group had Low-Level Laser Therapy (LLLT) two times a week for duration of six weeks, resulting in a total of 12 treatment sessions. During each session, the laser beam was administered according to a three-phase therapy regimen outlined as follows: (a) The pterygoid muscles’ insertion (b) during the second phase, radiation was applied to tender spots in the masseter and temporalis muscles that were discovered during first examination. (c) In the third phase, radiation was applied to the origin of the pterygoid muscles. The laser was applied for two minutes in each location throughout each phase, with a peak output of 500 mW and a wavelength of 810 nm. It was continuously administered, keeping a slight distance from the tissues. For target area, laser probe was positioned perpendicularly. It was necessary to calibrate the laser before use. Both the clinician and the patients wore protective eyewear. G**roup C**, was given threaded tapered screw appliance therapy (LLLT + TTSA) in addition to low-level laser therapy. TTSA was employed for the first time concurrently with the first LLLT session. No medicine was given to any of the groups during the trial. Analgesics or painkillers were not permitted for the patients to take.

The treatment plan was unknown to the one competent prosthodontist who did the whole pre- and post-clinical evaluations. It was advised to patients not to discuss therapy allocation with the examiner. At baseline (T0), which was thirty minutes before to the start of the therapy intervention, each patient had their visual analogue scale (VAS) pain score (0–10) assessed and their maximal mouth opening measured in millimeters. The results were as follows:

- Measurement of maximum mouth opening (MMO):

The maximum vertical distance in millimeters between the mandibular central incisor and the maxillary central incisor was measured at the midline while the patient’s jaw was actively opened using a millimeter ruler.

- VAS of pain: The patients used a visual analogue scale (VAS) to self-assess their level of pain, with 10 representing the most severe pain.
- Gothenburg Trismus Questionnaire (GTQ).

The GTQ seems to be the most reliable tool for measuring trismus that is now available since a more comprehensive assessment of trismus and its treatment effects requires a multidimensional, self-administered, trismus-specific measure, which is what the GTQ offers. Its wider applicability would allow for consistent recording of trismus-related issues and a chance to get patient feedback on treatment results. The updated GTQ version had 13 items spread over three domains: eating restriction (4 items), jaw-related issues (6 items), and muscular stress (3 items). Scores on the calculated scale vary from 0 to 100. Based on the symptom scales and items, a score of 100 represents the worst potential symptoms [25].

Measurements of maximal mouth opening (MMO) and visual analogue score (VAS) of pain were performed 5 times for each patient over the course of the trial (baseline, one, two weeks, one, three, and six months) in order to obtain quantitative data. Additionally, the time it took to return to normal was evaluated. If the following conditions were met, the normal state is considered to have been reached: (1) The pain VAS score was less than three (pain ˂ three); (2) The MMO measured 40 mm or more. (3) The GTQ scores were taken and near zero scores were considered normal.

The data was analyzed using the Statistical Package for the Social Sciences (SPSS Inc., Chicago, IL, USA), version 25. The Shapiro-Wilk test was utilized to determine whether the gathered data was normal, which indicated that the data followed a parametric and normally distributed pattern. Descriptive statistics, such as the mean and standard deviation, were used to summarize the data. The Tukey test and one-way analysis of variance (ANOVA) were utilized in a variety of measurements.Pairwise analysis was used to compare between each two groups. For every test used, results were deemed statistically significant if *P* ≤.050.

## Results

Thirty-six patients, ages 50 to 65, participated in this study. They were arbitrarily split up into three groups: Group A: was given threaded tapered screw appliance therapy (TTSA), 12 patients (eight males [66.7%] and four females [33.3%]), Group B: was given low-level laser therapy (LLLT), 12 patients (nine males [75%] and three females [25%]) and, Group C was given low-level laser therapy in addition to threaded tapered screw appliance therapy (LLLT + TTSA) 12 patients (seven males [58.3%] and five females [41.7%]).

**Fig. 5:**
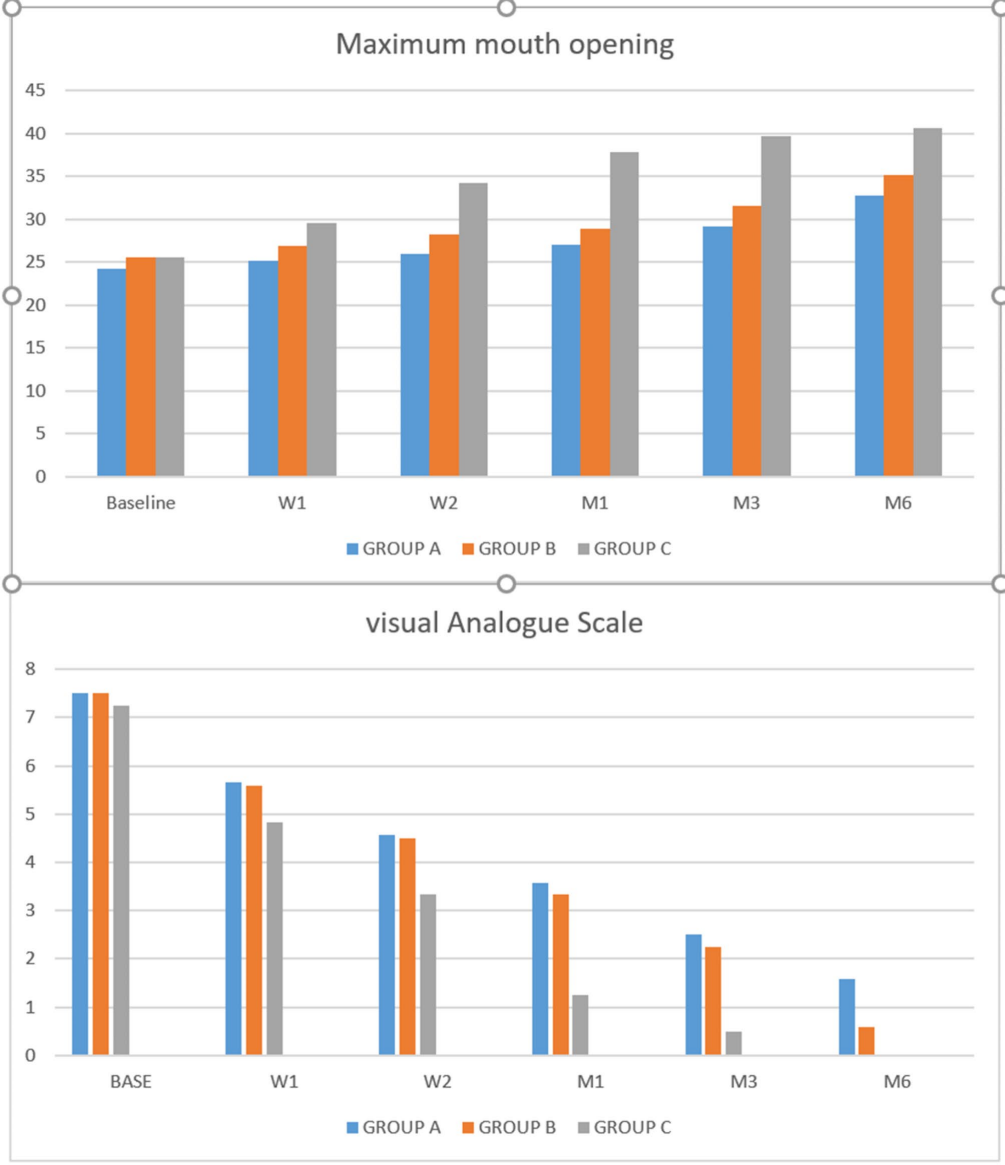
Comparison of visuale analogue scale and maximum mouth opening

Comparisons of VAS were presented in (Table 1), Fig. 5. At the baseline, there was no statistically significant between groups. At three months, there was no a statistically significant difference between group A-B but, there was a statistically significant difference between group A-C and group B-C. At the last six months, there was a statistically significant difference between groups. There was a statistically significant difference at different times of evaluation within all groups where (*P* <.0001).

**Table 1:** Comparison of visual analogue scale between and within groups

VAS	Base	W1	W2	M1	M3	M6	*P* value
	Mean ± sd	Mean ± sd	Mean ± sd	Mean ± sd	Mean ± sd	Mean ± sd	
Group A	7.50 ± 0.67 ^a^	5.67 ± 0.49^b^	4.58 ± 0.51^c^	3.58 ± 0.51^d^	2.50 ± 0.52^e^	1.58 ± 0.51^f^	< 0.0001
Group B	7.50 ± 0.52 ^a^	5.58 ± 0.51^b^	4.50 ± 0.52^c^	3.33 ± 0.65^d^	2.25 ± 0.62^e^	0.58 ± 0.51^f^	< 0.0001
Group C	7.25 ± 0.62 ^a^	4.83 ± 0.39^b^	3.33 ± 0.49^c^	1.25 ± 0.45^d^	0.50 ± 0.52^e^	0.00 ± 0.00^e^	< 0.0001
Group A-Group B	1.0000	0.6660	0.6916	0.2700	0.2798	< 0.0001	
Group A-Group C	0.3222	0.0001	< 0.0001	< 0.0001	< 0.0001	< 0.0001	
Group B-Group C	0.3222	0.0004	< 0.0001	< 0.0001	< 0.0001	0.0018	

a-f = means with the same small letter in each row are not significantly different at *p* ≤.05 using Tukey test

For Comparisons of MMO were presented in (Table 2), Fig. 5 At the baseline, there was a statistically significant between group A-B and group A-C but, there was no statistically significant between group B-C. At three and six months, there was a statistically significant difference between all groups. There was a statistically significant difference at different times of evaluation within all groups where (*P* <.0001).

**Table 2:** Comparison of maximum mouth opening between and within groups

MMO	Base	W1	W2	M1	M3	M6	*P* value
	Mean ± sd	Mean ± sd	Mean ± sd	Mean ± sd	Mean ± sd	Mean ± sd	
Group A	24.17 ± 0.72^e^	25.17 ± 0.58^d^	25.92 ± 1.00^d^	27.08 ± 1.00^c^	29.17 ± 1.34^b^	32.75 ± 1.60^a^	< 0.0001
Group B	25.58 ± 0.90^e^	26.92 ± 0.90^d^	28.25 ± 0.87^c^	28.92 ± 1.08^c^	31.58 ± 1.24^b^	35.17 ± 1.27^a^	< 0.0001
Group C	25.58 ± 0.79^e^	29.58 ± 0.90^d^	34.17 ± 1.03^c^	37.83 ± 1.27^b^	39.75 ± 1.06^a^	40.58 ± 0.79^a^	< 0.0001
Group A-Group B	0.0001	< 0.0001	< 0.0001	0.0003	< 0.0001	< 0.0001	
Group A-Group C	0.0001	< 0.0001	< 0.0001	< 0.0001	< 0.0001	< 0.0001	
Group B-Group C	1.0000	< 0.0001	< 0.0001	< 0.0001	< 0.0001	< 0.0001	

f = means with the same small letter in each row are not significantly different at *p* ≤.05 using Tukey test

(Table 3), showed the descriptive analysis of Gothenburg Trismus Questionnaire (GTQ) include the three domains: jaw-related issues (6 items) stiffness in jaw, aches in face, pain in moving jaw (opening mouth/chewing), problems opening mouth wide, pain or soreness in jaw muscles, problems yawning. Eating restriction (4 items) eat solid food, put food in mouth, eat soft food, bite off. And muscular stress (3 items) clench your teeth, press with your tongue, noises from jaw, which were expressed in Mean ± SD.

**Table 3:** The descriptive analysis of Gothenburg Trismus Questionnaire (GTQ)

	Base	w1	w2	m1	m3	m6	
**Stiffness in jaw**	Mean ± SD	Mean ± SD	Mean ± SD	Mean ± SD	Mean ± SD	Mean ± SD	
**Group A**	71.33 ± 2.31^a^	65.08 ± 2.07 ^b^	56.75 ± 2.56 ^c^	42.92 ± 2.39 ^d^	35.00 ± 2.73 ^e^	21.75 ± 2.93 ^f^	
**Group B**	71.83 ± 2.69 ^a^	62.75 ± 2.05 ^b^	52.00 ± 2.52 ^c^	33.75 ± 3.17 ^d^	28.58 ± 3.15 ^e^	17.58 ± 2.35 ^f^	
**Group C**	73.00 ± 2.37 ^a^	56.92 ± 2.47 ^b^	48.33 ± 2.84 ^c^	32.25 ± 2.18 ^d^	19.08 ± 2.19 ^e^	8.92 ± 2.23 ^f^	
**Group A–Group B**	0.6224	0.0140	0.0001	< 0.0001	< 0.0001	0.0003	
**Group A–Group C**	0.1070	< 0.0001	< 0.0001	< 0.0001	< 0.0001	< 0.0001	
**Group B–Group C**	0.2544	< 0.0001	0.0018	0.1691	< 0.0001	< 0.0001	
**Aches in face and jaw**							
**Group A**	72.25 ± 2.77 ^a^	65.25 ± 2.99 ^b^	57.00 ± 2.00 ^c^	42.67 ± 2.06 ^d^	34.75 ± 2.30 ^e^	21.08 ± 1.78 ^f^	
**Group B**	73.33 ± 2.35 ^a^	62.50 ± 2.43 ^b^	51.92 ± 2.39 ^c^	34.33 ± 2.99 ^d^	25.75 ± 3.05 ^e^	16.92 ± 3.34 ^f^	
**Group C**	73.00 ± 2.66 ^a^	57.00 ± 3.02 ^b^	46.00 ± 2.41 ^c^	31.83 ± 1.34 ^d^	19.58 ± 2.19 ^e^	8.58 ± 2.23 ^f^	
**Group A–Group B**	0.3147	0.0230	< 0.0001	< 0.0001	< 0.0001	0.0003	
**Group A -Group C**	0.4846	< 0.0001	< 0.0001	< 0.0001	< 0.0001	< 0.0001	
**Group B–Group C**	0.7554	< 0.0001	< 0.0001	0.0099	< 0.0001	< 0.0001	
**Pain in moving jaw**							
**Group A**	73.58 ± 3.85 ^a^	65.67 ± 2.77 ^b^	57.17 ± 2.55 ^c^	42.25 ± 2.26 ^d^	34.50 ± 2.58 ^e^	20.83 ± 2.17 ^f^	
**Group B**	73.25 ± 2.22 ^a^	62.58 ± 2.68 ^b^	52.42 ± 2.68 ^c^	34.17 ± 3.07 ^d^	27.42 ± 3.20 ^e^	18.00 ± 2.59 ^f^	
**Group C**	72.17 ± 2.59 ^a^	55.42 ± 3.50 ^b^	45.33 ± 3.98 ^c^	31.75 ± 1.96 ^d^	20.08 ± 1.83 ^e^	9.42 ± 2.02 ^f^	
**Group A–Group B**	0.7850	0.0171	0.0008	< 0.0001	< 0.0001	0.0045	
**Group A–Group C**	0.2508	< 0.0001	< 0.0001	< 0.0001	< 0.0001	< 0.0001	
**Group B–Group C**	0.3779	< 0.0001	< 0.0001	0.0226	< 0.0001	< 0.0001	
**Problems opening mouth**							
**Group A**	72.67 ± 2.67 ^a^	65.17 ± 3.54 ^b^	59.00 ± 3.93 ^c^	41.92 ± 2.35 ^d^	34.33 ± 2.77 ^e^	20.25 ± 2.01 ^f^	
**Group B**	73.92 ± 2.31 ^a^	62.75 ± 2.30 ^b^	51.75 ± 2.45 ^c^	34.25 ± 2.45 ^d^	26.67 ± 3.80 ^e^	17.92 ± 3.26 ^f^	
**Group C**	73.50 ± 2.71^a^	56.17 ± 3.41 ^b^	46.42 ± 4.44 ^c^	32.33 ± 2.15 ^d^	19.33 ± 2.02 ^e^	7.67 ± 2.77 ^f^	
**Group A–Group B**	0.2427	0.0675	< 0.0001	< 0.0001	< 0.0001	0.0440	
**Group A–Group C**	0.4334	< 0.0001	< 0.0001	< 0.0001	< 0.0001	< 0.0001	
**Group B–Group C**	0.6942	< 0.0001	0.0013	0.0513	< 0.0001	< 0.0001	
**Pain in jaw muscle**							
**Group A**	73.42 ± 2.23 ^a^	65.58 ± 3.40 ^b^	55.08 ± 3.50 ^c^	42.00 ± 2.56 ^d^	35.25 ± 2.86 ^e^	20.25 ± 1.82 ^f^	
**Group B**	73.08 ± 2.07 ^a^	64.42 ± 3.23 ^b^	51.92 ± 2.75 ^c^	33.75 ± 3.62 ^d^	26.08 ± 3.09 ^e^	17.17 ± 3.61 ^f^	
**Group C**	73.67 ± 3.17 ^a^	55.17 ± 4.49 ^b^	43.83 ± 3.04 ^c^	32.08 ± 2.78 ^d^	20.00 ± 2.26 ^e^	8.42 ± 1.93 ^f^	
**Group A- Group B**	0.7497	0.4512	0.0179	< 0.0001	< 0.0001	0.0063	
**Group A- Group C**	0.8108	< 0.0001	< 0.0001	< 0.0001	< 0.0001	< 0.0001	
**Group B- Group C**	0.5772	< 0.0001	< 0.0001	0.1858	< 0.0001	< 0.0001	
**Problem in yawing**	Mean ± sd	Mean ± sd	Mean ± sd	Mean ± sd	Mean ± sd	Mean ± sd	
**Group A**	73.42 ± 2.43^a^	64.75 ± 2.63^b^	56.25 ± 2.77^c^	42.25 ± 1.48 ^d^	34.33 ± 3.08 ^e^	21.33 ± 2.02 ^f^	< 0.0001
**Group B**	72.67 ± 2.84 ^a^	62.92 ± 2.11 ^b^	52.58 ± 2.11 ^c^	34.25 ± 2.42 ^d^	27.17 ± 2.59 ^e^	18.00 ± 2.09 ^f^	< 0.0001
**Group C**	*73.08 ± 2.39* ^*a*^	*56.67 ± 1.83* ^*b*^	*46.58 ± 2.75* ^*c*^	*31.92 ± 2.39* ^*d*^	*20.83 ± 1.99* ^*e*^	*9.83 ± 1.75* ^*f*^	*< 0.0001*
**Group A-Group B**	**0.4783**	**0.0507**	**0.0013**	**< 0.0001**	**< 0.0001**	**0.0002**	
**Group A-Group C**	0.7519	< 0.0001	< 0.0001	< 0.0001	< 0.0001	< 0.0001	
**Group B-Group C**	0.6929	< 0.0001	< 0.0001	0.0117	< 0.0001	< 0.0001	
**Eat solid food**							
**Group A**	73.83 ± 2.86^a^	64.92 ± 2.91^b^	56.33 ± 3.52^c^	42.75 ± 2.67^d^	33.58 ± 2.71^e^	20.50 ± 2.15^f^	< 0.0001
**Group B**	73.83 ± 2.89^a^	63.67 ± 2.53^b^	52.67 ± 2.61^c^	34.00 ± 2.66^d^	26.92 ± 2.07^e^	18.00 ± 2.13^f^	< 0.0001
**Group C**	73.92 ± 3.23^a^	56.25 ± 1.96^b^	46.58 ± 2.68^c^	32.08 ± 2.23^d^	19.83 ± 1.99^e^	8.92 ± 2.27 ^f^	< 0.0001
**Group A-Group B**	1.0000	0.2289	0.0047	< 0.0001	< 0.0001	0.0085	
**Group A-Group C**	0.9461	< 0.0001	< 0.0001	< 0.0001	< 0.0001	< 0.0001	
**Group B-Group C**	0.9461	< 0.0001	< 0.0001	0.0724	< 0.0001	< 0.0001	
**Put food in mouth**							
**Group A**	73.25 ± 2.67^a^	65.75 ± 3.28^b^	55.83 ± 3.27^c^	42.33 ± 2.46^d^	34.00 ± 2.70^e^	20.50 ± 1.83^f^	< 0.0001
**Group B**	74.00 ± 2.73^a^	62.83 ± 2.17^b^	52.50 ± 2.02^c^	33.42 ± 2.81^d^	26.25 ± 3.52^e^	18.50 ± 2.54^f^	< 0.0001
**Group C**	73.50 ± 2.84^a^	56.50 ± 2.75^b^	46.83 ± 2.33^c^	31.83 ± 1.90^d^	19.58 ± 2.27^e^	9.42 ± 1.62^f^	< 0.0001
**Group A–Group B**	0.5085	0.0145	0.0035	< 0.0001	< 0.0001	0.0219	
**Group A–Group C**	0.8250	< 0.0001	< 0.0001	< 0.0001	< 0.0001	< 0.0001	
**Group B–Group C**	0.6588	< 0.0001	< 0.0001	0.1185	< 0.0001	< 0.0001	
**Eat soft food**							
**Group A**	73.67 ± 3.08^a^	65.58 ± 2.50^b^	56.50 ± 2.54^c^	42.58 ± 1.88^d^	34.75 ± 2.42^e^	20.92 ± 2.15^f^	< 0.0001
**Group B**	74.08 ± 2.91^a^	62.58 ± 1.98^b^	53.08 ± 2.81^c^	34.08 ± 3.12^d^	26.25 ± 2.67^e^	18.17 ± 1.99^f^	< 0.0001
**Group C**	73.92 ± 2.81^a^	56.42 ± 2.94^b^	46.08 ± 3.26^c^	31.75 ± 1.71^d^	20.00 ± 2.37^e^	9.00 ± 1.76^f^	< 0.0001
**Group A–Group B**	0.7303	0.0060	0.0066	< 0.0001	< 0.0001	0.0017	
**Group A–Group C**	0.8361	< 0.0001	< 0.0001	< 0.0001	< 0.0001	< 0.0001	
**Group B–Group C**	0.8903	< 0.0001	< 0.0001	0.0193	< 0.0001	< 0.0001	
**Bite_off**							
**Group A**	74.17 ± 3.27^a^	65.92 ± 2.64^b^	56.08 ± 3.75^c^	42.50 ± 1.93^d^	33.92 ± 3.00^e^	21.17 ± 1.75^f^	< 0.0001
**Group B**	73.83 ± 2.55^a^	63.00 ± 2.70^b^	52.50 ± 1.93^c^	34.83 ± 2.79^d^	26.92 ± 2.87^e^	18.08 ± 3.32^f^	< 0.0001
**Group C**	73.50 ± 2.88^a^	56.67 ± 2.46^b^	46.75 ± 1.86^c^	31.50 ± 2.11^d^	19.75 ± 2.34^e^	8.75 ± 1.96^f^	< 0.0001
**Group A–Group B**	0.7811	0.0097	0.0024	< 0.0001	< 0.0001	0.0040	
**Group A–Group C**	0.5791	< 0.0001	< 0.0001	< 0.0001	< 0.0001	< 0.0001	
**Group B–Group C**	0.7811	< 0.0001	< 0.0001	0.0012	< 0.0001	< 0.0001	
**Clench your teeth**							
**Group A**	73.25 ± 2.49^a^	65.67 ± 3.14^b^	56.33 ± 3.03^c^	42.50 ± 2.11^d^	34.25 ± 2.18^e^	20.83 ± 2.66^f^	< 0.0001
**Group B**	74.17 ± 2.52^a^	62.58 ± 2.19^b^	52.08 ± 2.02^c^	34.58 ± 3.12^d^	26.08 ± 3.42^e^	18.58 ± 2.35^f^	< 0.0001
**Group C**	73.42 ± 2.75^a^	55.92 ± 2.43^b^	46.33 ± 2.39^c^	31.92 ± 1.88^d^	20.08 ± 2.31^e^	8.92 ± 2.07^f^	< 0.0001
**Group A–Group B**	0.3917	0.0069	0.0002	< 0.0001	< 0.0001	0.0264	
**Group A–Group C**	0.8756	< 0.0001	< 0.0001	< 0.0001	< 0.0001	< 0.0001	
**Group B–Group C**	0.4826	< 0.0001	< 0.0001	0.0112	< 0.0001	< 0.0001	
**press with your tongue**							
**Group A**	73.83 ± 2.82^a^	65.92 ± 2.81^b^	56.08 ± 2.68^c^	42.25 ± 1.96^d^	35.08 ± 1.93^e^	20.33 ± 2.15^f^	< 0.0001
**Group B**	73.08 ± 2.81^a^	63.25 ± 1.86^b^	52.83 ± 2.21^c^	34.00 ± 2.98^d^	25.75 ± 2.56^e^	17.92 ± 2.35^f^	< 0.0001
**Group C**	73.50 ± 2.81^a^	55.83 ± 2.79^b^	45.42 ± 3.23^c^	32.17 ± 2.44^d^	20.33 ± 2.46^e^	8.83 ± 2.29^f^	< 0.0001
**Group A–Group B**	0.5186	0.0144	0.0065	< 0.0001	< 0.0001	0.0134	
**Group A–Group C**	0.7736	< 0.0001	< 0.0001	< 0.0001	< 0.0001	< 0.0001	
**Group B–Group C**	0.7193	< 0.0001	< 0.0001	0.0814	< 0.0001	< 0.0001	
**Noise from jaw**							
**Group A**	73.83 ± 2.25^a^	65.50 ± 2.02^b^	55.83 ± 2.66^c^	42.00 ± 1.71^d^	34.17 ± 2.21^e^	20.17 ± 2.82^f^	< 0.0001
**Group B**	74.17 ± 2.79^a^	63.58 ± 2.50^b^	52.58 ± 1.83^c^	35.00 ± 3.19^d^	27.25 ± 2.73^e^	17.67 ± 3.55^f^	< 0.0001
**Group C**	74.00 ± 2.26^a^	55.50 ± 3.18^b^	45.83 ± 2.37^c^	31.33 ± 1.50^d^	20.00 ± 2.63^e^	9.25 ± 2.01^f^	< 0.0001
**Group A–Group B**	0.5421	0.2289	0.0053	< 0.0001	< 0.0001	0.0253	
**Group A–Group C**	0.4826	< 0.0001	< 0.0001	< 0.0001	< 0.0001	< 0.0001	
**Group B–Group C**	0.87459	< 0.0001	< 0.0001	0.0182	< 0.0001	< 0.0001	

a-f = means with the same small letter in each row are not significantly different at *p* ≤.05 using Tukey test

At baseline, the highest mean values for GTQ items were noted. These values demonstrated a progressive decline across all groups, with a statistically significant difference between assessment times for every items in GTQ within each group (*P* <.0001). Group C recorded the least values for GTQ symptoms followed by group B followed by group A. For example, (9.83 ± 1.75), (18.00 ± 2.09), (21.33 ± 2.02) respectively in problems yawning item at the last six months of evaluation period. At the baseline, there was no statistically significant between groups in different items. At three and six months, there was a statistically significant difference between groups in different items.

At baseline, the highest mean values for GTQ were noted. These values demonstrated a progressive decline across all groups, with a statistically significant difference between assessment times for every domain in GTQ within each group (*P* <.001). Group C recorded the least values for GTQ symptoms followed by group B followed by group A. For example, (9.83 ± 1.75), (18.00 ± 2.09), (21.33 ± 2.02) respectively in problems yawning item at the last six months of evaluation period.

## Discussion

A statistically significant difference was found between the evaluated groups, according to the results. supporting the study hypothesis that the combination of TTSA and LLLT would reduce clinical signs and symptoms more than applying either TTSA or LLLT alone. Nevertheless, the combination therapy was found to be no more effective than either laser therapy or appliance therapy, refuting the null hypothesis.

Three conservative therapies have been assessed in this study for patients who have radiation induced trismus (RIT). Conservative and surgical therapy were shown to be able to accomplish the same goals for patients in RIT in a prior study [4]. This study’s goal was spurred by the awareness that improving the conservative approach can help prevent the necessity for invasive surgical treatments and the related complications.

In this study, the visual analogue scale (VAS) was employed to measure pain quantitatively and identify changes in pain that are clinically significant. It is a legitimate and trustworthy tool [26]. Measures of vertical range of motion have previously shown excellent reliability [27]. According to the study’s findings, every study patient significantly improved their VAS and MMO scores at the majority of evaluation times when compared to the baseline, which had the highest mean value. For the three groups, early in the first week, there was an improvement. nevertheless, group C shown a notable improvement. Regarding, maximum mouth opening, across all groups, there was a statistically significant difference at various evaluation times where (*P* <.0001) but, for group C, the improvement began early in the first week, and for groups A and B, it began at three months. These findings were consistent with a prior investigation that used low intensity ultrasound (LIUS), traditional exercise therapy (TET), low level laser treatment (LLLT), and TET to assess the effects of temporomandibular joint (TMJ) discomfort and trismus following recovery from head and neck cancer (HNC) [24]. Also, another study reported an improvement in the maximum mouth opening after using (TTSA) to manage the symptoms of trismus. The idea of employing an acrylic resin screw that has been digitally developed and is threaded and tapered may work because it is straightforward for patients to use, simple and affordable in comparison to other ways. By acting on the patient’s depressor group of muscles, the threaded tapered acrylic screw helps to separate their jaws. The success of this type of appliance primarily depends on patient motivation.

Furthermore, trismus was treated with LLLT in this clinical trial following recovery from head and neck cancer (HNC). The findings of this study indicate that LLLT increases maximum mouth opening and facilitates pain alleviation. Similar findings were found in a study by Zecha et al., [28] which reported that LLLT is highly beneficial in alleviating side effects following HNC chemotherapy and radiation therapy, including trismus, dysgeusia, dysphagia, lymphedema, and speech impairments. Several authors [29–31] found that LLLT is useful in lowering pain and increasing range of motion, which is consistent with our findings. The benefits of laser therapy are numerous and include biostimulation, temperature-neutral analgesic effects, vasodilatation, edema reduction, cellular metabolism elevation, enhanced pain threshold resulting from changes in cell membrane structure, and wound healing duration. In contrast to the current study, another studies [32–34] revealed that LLLT is ineffective in treating trismus, oedema, and pain in myofascial pain syndrome. Divergent equipment brands, application sites, assessment and classification criteria, and variations in the characteristics and quantities employed could have contributed to the discrepancies in the results. This study was distinctive in that it addressed the management of trismus using both intraoral and extraoral laser therapy. The literature contains varying application points for LLLT. There was seldom any intra-oral application seen, however the application sites are typically through the skin that covers the affected muscles and TMJ. Intraoral applications were carried out in this investigation using a special probe from a laser device. According to Ahmad et al. [35], deeper-seated tissues can be treated with the laser beam utilized in this work because its wavelength of 810 nm allows for more penetration.

The GTQ has been proposed as a screening tool for jaw-related problems, muscle strain, and feeding restrictions [36]. Additionally, The GTQ has been suggested as a tool to assess rehabilitation and jaw physical therapy outcomes [37]. In the current study, the greatest mean values for GTQ were found at baseline. These values demonstrated a progressive decline across all groups, with a statistically significant difference between assessment times for every domain in GTQ within each group. Group C recorded the least values for GTQ symptoms followed by group B followed by group A. When comparing health-related quality of life in the domains of mouth opening, jaw-related issues, feeding restraints, and muscular tension, the combination of TTSA and LLLT yielded the best results (*P* <.0001). The beneficial benefits of LLLT on pain and spasms in the muscles and the ability of TTSA to act on the patient’s depressor group of muscles by raising their maximal mouth and restoring normal function could both be explained by the fact that both therapies increase vasodilation.

In summary, all available therapy modalities have the potential to effectively improve radiation induced trismus; however, the combination of TTSA and LLLT group appears to yield the most rapid and optimal enhancement. The study’s clinical implications include the recommendation that low-level laser therapy and threaded tapered screw appliance therapy (TTSA) can be used in conjunction for effective treatment of radiation induced trismus (RIT).

Patient population used in this study is considered small sample size and it is one of the study limitations.Also, other confounding factors are one of the study limitation so, further studies are needed to be done with large sample size and including the other confounding factors.

## Conclusion

Considering the findings of this research, TTSA, LLLT, and combined therapies are effective ways to alleviate trismus and discomfort in people who have had HNC. Although the study had a limited sample size, it demonstrated that the combination LLLT and TTSA was more effective than either TTSA alone or LLLT in treating trismus after HNC. As such, it may be used as a complementary treatment for these conditions.

## Data Availability

The datasets used in the current study are available from the corresponding author upon request.
